# Expanded‐Access Use of Elamipretide Improves Quality of Life in Patients With Rare Mitochondrial Disorders Characterized by Ophthalmic Symptoms: A Case Series

**DOI:** 10.1002/ccr3.9591

**Published:** 2024-11-29

**Authors:** Sharique Ansari, Mary Kay Koenig

**Affiliations:** ^1^ Kane Hall Barry Neurology Bedford Texas USA; ^2^ Department of Pediatrics, Division of Child & Adolescent Neurology, Center for the Treatment of Pediatric Neurodegenerative Disease University of Texas McGovern Medical School Houston Texas USA

**Keywords:** Cardiolipin, CPEO, Elamipretide, mitochondrial disease, NARP syndrome

## Abstract

This case series presents the use of elamipretide in two patients with different progressive mitochondrial disorders (chronic progressive external ophthalmoplegia [CPEO] plus and neuropathy, ataxia, and retinitis pigmentosa [NARP] syndrome) characterized by ophthalmic traits. Elamipretide was well tolerated and both patients demonstrated improvement in symptoms while on therapy.

## Graphical abstract

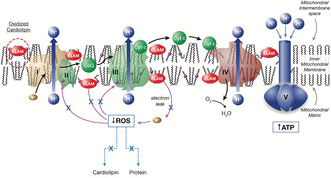

## 1 Introduction

Chronic progressive external ophthalmoplegia (CPEO) plus and neuropathy, ataxia, and retinitis pigmentosa (NARP) syndrome, caused by defects of mitochondrial energy production, are progressive neurodegenerative disorders presenting with ophthalmic symptoms [1, 2]. While progress has been made in the therapeutic pipeline for some mitochondrial diseases, no United States Food and Drug Administration (FDA)‐approved therapies exist for these conditions. The FDA has provided guidance on expanded access for the use of investigational drugs in patients with serious diseases who lack therapeutic alternatives when the potential benefits of treatment outweigh the risks [3]. Elamipretide is a cell‐permeable peptide that targets the inner mitochondrial membrane where it binds to cardiolipin, resulting in improved membrane stability, enhanced adenosine triphosphate (ATP) synthesis, and reduced reactive oxygen species (ROS) production [4, 5]. This investigational drug is being developed for the treatment of patients with a variety of mitochondrial diseases, including many characterized by ophthalmic disorders, as elamipretide has demonstrated promising results in patients with primary mitochondrial myopathy (PMM) [6] and Barth syndrome [7]. In this series, we present the use of elamipretide under an Expanded Access Program (EAP) in two patients with different mitochondrial disorders consisting of ophthalmic components, with a goal of providing further support for the use of elamipretide in mitochondrial diseases.

## 2 Case History/Examination

### 2.1 Patient 1

A 67‐year‐old male presented with neurodegenerative symptoms including hearing loss, abnormal gait and balance, lower extremity paresthesia and weakness, muscular atrophy, incoordination, dysphagia, depression, extreme fatigue, cognitive decline, and Parkinsonism. His pertinent family history consisted of a sister who experienced similar symptoms prior to her death from respiratory complications at 74 years of age, although her disease was never formally diagnosed. He also reported a history of tremors in his father and brother. The patient reported a history of bilateral ptosis and progressive ophthalmoplegia since his early teenage years; however, he was otherwise healthy through adolescence and early adulthood, competing in sports through college. As his eye issues worsened, the patient underwent strabismus surgery and two ptosis surgeries between the ages of 25 and 42 years. In his 40s, his symptoms progressed beyond eye issues to include loss of balance, frequent tripping, and neuropathy. Eventually, he was unable to participate in cross‐country skiing races or run for exercise. At this point in his life, he was misdiagnosed with Kearns‐Sayre syndrome at a different clinic based on symptoms alone. The patient reluctantly retired at 62 years of age due to symptoms interfering with his ability to perform the duties required in his marketing career. The patient also lost his ability to drive a vehicle.

Upon presentation to our clinic, his more recent progressive symptoms included stuttering and speech problems, dysphagia, and short‐term memory issues. In addition, the patient has a history of arrythmias and cardiac ablation. He underwent genetic testing which revealed a diagnosis of CPEO secondary to a heterozygous pathogenic variant (c.1879C>T) in *POLG* and a variant of unknown significance (c.3301G>A) in *POLG*. Of note, genetic testing for oculopharyngeal muscular dystrophy was negative. Physical examination revealed bilateral temporal muscle atrophy, facial weakness, ptosis, fixed ophthalmoplegia with weakness of ocular muscles, areflexia of the bilateral lower extremities, muscular contracture of the elbows, proximal weakness, ataxia, sustentation tremor, bilateral foot drop, and distal sensory loss. Remarkable laboratory values included creatine kinase 740 U/L (reference interval 24–204 U/L), aldolase 11.3 U/L (reference interval 3.3–10.3 U/L), myoglobin 173 ng/mL (reference interval 28–72 ng/mL), and vitamin B12 ≥ 2000 pg/mL (reference interval 232–1245 pg/mL). Recent magnetic resonance imaging (MRI) of his brain revealed mild chronic white matter ischemic changes with no other intracranial abnormalities. In addition, a recent nerve conduction study showed electrodiagnostic evidence of moderate, generalized, sensorimotor, axonal peripheral polyneuropathy affecting the lower extremities. Evidence of mild carpal tunnel in the left wrist was also noted. Nerve conduction studies (NCS) revealed symmetrical, length‐dependent, primarily axonal distal neuropathy without conduction block or temporal dispersion and needle electromyography (EMG) showed chronic denervation in distal foot muscle, suggesting length‐dependent process. His medications consisted of omeprazole 40 mg daily, propranolol 10 mg twice daily, gabapentin 300 mg twice daily, duloxetine 30 mg twice daily, baby aspirin, and various over‐the‐counter vitamins and supplements.

### 2.2 Patient 2

A 40‐year‐old male with a primary diagnosis of NARP syndrome due to a 91.2% heteroplasmic mutation at m.8993T>G presented with primary symptoms of retinitis pigmentosa, sensorineural hearing loss, progressive cerebellar ataxia, cognitive impairment, long‐term memory loss, left eye exotropia, and proximal myopathy. His visual compromise was first noted prior to 2 years of age, and the patient had been legally blind since 14 years of age, when he was formally diagnosed with NARP syndrome. The patient developed sensorineural hearing loss at 20 years of age and used hearing aids. In addition, he had a history of slight motor and cognitive delays as well as slow growth during childhood. An MRI of his brain demonstrated findings consistent with Leigh syndrome, including atrophy of the cerebellum and brainstem with bilateral cystic changes in the basal ganglia. Aside from amlodipine for hypertension, his only medications included a variety of over‐the‐counter vitamins and supplements. Eye examination revealed left eye exotropia, bilateral ptosis, anisocoric pupils, and absent vision with the ability to see some light in the right eye. Physical examination showed immature and moderately dysarthric speech, delayed cognition, decreased sensation in distal extremities, absent deep tendon reflexes, decreased proximal muscle strength, and pes cavus with toes upgoing bilaterally. The patient was able to rise from the chair independently, but he was unsteady and braced himself on the chair. He was noted to walk with his feet everted with an unsteady gait.

The patient initially received elamipretide therapy at 40 mg/day subcutaneously (SC) for approximately 10 months through participation in a clinical trial (SPIMM‐301, Stealth BioTherapeutics). During the time of treatment, the patient experienced an increased ability to work and socialize as well as improvement in exercise ability, balance, and posture. Within the first 6 months of treatment, his walking capacity on the 6‐minute walk test (6MWT) improved from 120 to 259 m. In addition, the patient demonstrated improved fatigue and quality of life (QOL) scores. After the trial ended, within approximately 4 months of elamipretide cessation, the patient and his family reported loss of any improvements made during treatment. Specifically, they noted worsened balance, orientation, mobility, weakness, lethargy, and confusion. The patient lost most independence and could no longer be left home alone. He was unable to ambulate, could not hold his head up, and drooled.

## 3 Methods

### 3.1 Patient 1

Due to his declining condition, the patient was enrolled in the intermediate‐size EAP (SPIES‐006, Stealth BioTherapeutics) for SC elamipretide administration in patients with genetically confirmed rare diseases with known mitochondrial dysfunction. Prior to beginning treatment with elamipretide, patient consent was obtained, and drug administration was approved by the FDA and local Institutional Review Board (IRB). Elamipretide was initiated at a dose of 40 mg/day (0.5 mL) injected SC. While his gabapentin dose was increased for worsening neuropathy and primidone was added for tremors, no other medication changes were made during the elamipretide treatment period.

### 3.2 Patient 2

Approximately  months after his last dose of elamipretide through the clinical trial, the patient was enrolled into the EAP (SPIES‐006, Stealth BioTherapeutics) for SC elamipretide and reinitiated treatment with daily elamipretide at 40 mg SC. Prior to beginning treatment with elamipretide or changing elamipretide doses, patient consent was obtained, and drug administration was approved by the FDA and the local IRB. After about 1 year on elamipretide treatment through the EAP, the daily dose was increased to 60 mg SC to further optimize response.

## 4 Conclusion and Results

### 4.1 Patient 1

Upon his first injection, the patient reported an immediate sensation in his leg that radiated throughout his body. Within hours of the first injection, the patient felt tension in his eyes as if they were regaining motion. After only 2 weeks of treatment, the patient could raise his eyebrows and look upward, ambulated more quickly, and experienced fewer head tremors. Within 1 month of beginning elamipretide, the patient and his family reported significant improvement in stamina, muscle strength, balance, posture, and stability. He was able to stand without assistance, and his eyes were no longer fixed in place. The patient reported walking and riding his bicycle weekly as well as lifting heavier weights at the gym. After about 3 months of daily elamipretide therapy, the patient went cross‐country skiing at 9400 ft elevation, an activity he was unable to perform in over 5 years. The patient hiked over six miles at high elevation in Yosemite National Park after five consecutive months of elamipretide treatment. In addition, the patient was able to enjoy other leisure activities, including yardwork and playing with his young grandchildren.

The patient continued to improve after 1 year on elamipretide therapy. The patient increased his biking distance from 12 miles (8 months on elamipretide) to 15 miles (16 months on elamipretide). Despite recently developing rotoscoliosis in his lower back and kyphosis in his upper back, after about 18 months on elamipretide therapy, the patient continued to report drastic improvements in his strength, endurance, and overall quality of life. The patient was able to ride his bicycle for 20 miles in the heat twice weekly, lift heavier weights, and ambulate for five miles. He noted gaining independence and caring for his toddler grandchild. Despite vast improvement in many symptoms while receiving elamipretide, his dysphagia persisted. During the duration of elamipretide therapy, the only adverse reactions reported were mild injection site reactions, and routine laboratory values (complete blood count and comprehensive metabolic panel) remained stable.

### 4.2 Patient 2

The patient's family reported decreased ataxia and increased ambulation distance (up to 50 yards) after only 3 months of treatment. They also noted reduced dysarthria, improved ability to perform ADLs, and increased independence. His family again felt comfortable leaving the patient at home alone. Physical examination revealed improved strength and ambulation, such that the patient stood easily from the chair and ambulated steadily with minimal assistance. His feet were everted when standing, and ataxia remained. His creatine kinase trended down from 563 U/L pre‐elamipretide dosing through the EAP to 255 U/L with daily elamipretide treatment for approximately 11 months.

When the daily dose was increased to 60 mg SC after about 1 year in the EAP, a dramatic improvement in his condition was observed within 6 months of this dose increase and included resolution of previous walking problems, return of long‐term memory, and improvement in QOL scores. The patient and his family reported increases in his energy, stamina, walking ability, and distance. They also reported improvement in his hearing, speech clarity, balance, strength, and endurance. The patient could engage in more in‐depth conversations and showed more metered responses to accidents. He was able to participate in more leisure activities, including gardening, golfing, and traveling. Upon physical examination, the patient stood easily from the chair without sway or ataxia. He kept his feet in a normal, forward‐facing position and ambulated steadily with no assistance. The patient spoke mostly in single words but was understandable. The patient demonstrated 5/5 muscle strength to his upper and lower extremities and was able to hold his head up well. Since his increase in elamipretide dose, his creatine kinase decreased from 255 to 139 U/L and has remained stable. Ten months after the dose increase, the patient competed in the Special Olympics track event for the first time in over 7 years. Specifically, the patient participated in the javelin throw and the 25‐m walk. Eighteen months after the dose increase, the patient reported some return of vision. Specifically, the patient admitted to seeing colors, but due to his cognitive impairment, more specific or detailed information regarding his visual improvements could not be obtained. During the duration of elamipretide treatment, mild injection site reactions were the only adverse event reported. No other medication changes were made, and routine laboratory values (complete blood count and comprehensive metabolic panel) remained stable during the treatment period.

## 5 Discussion

**FIGURE 1:**
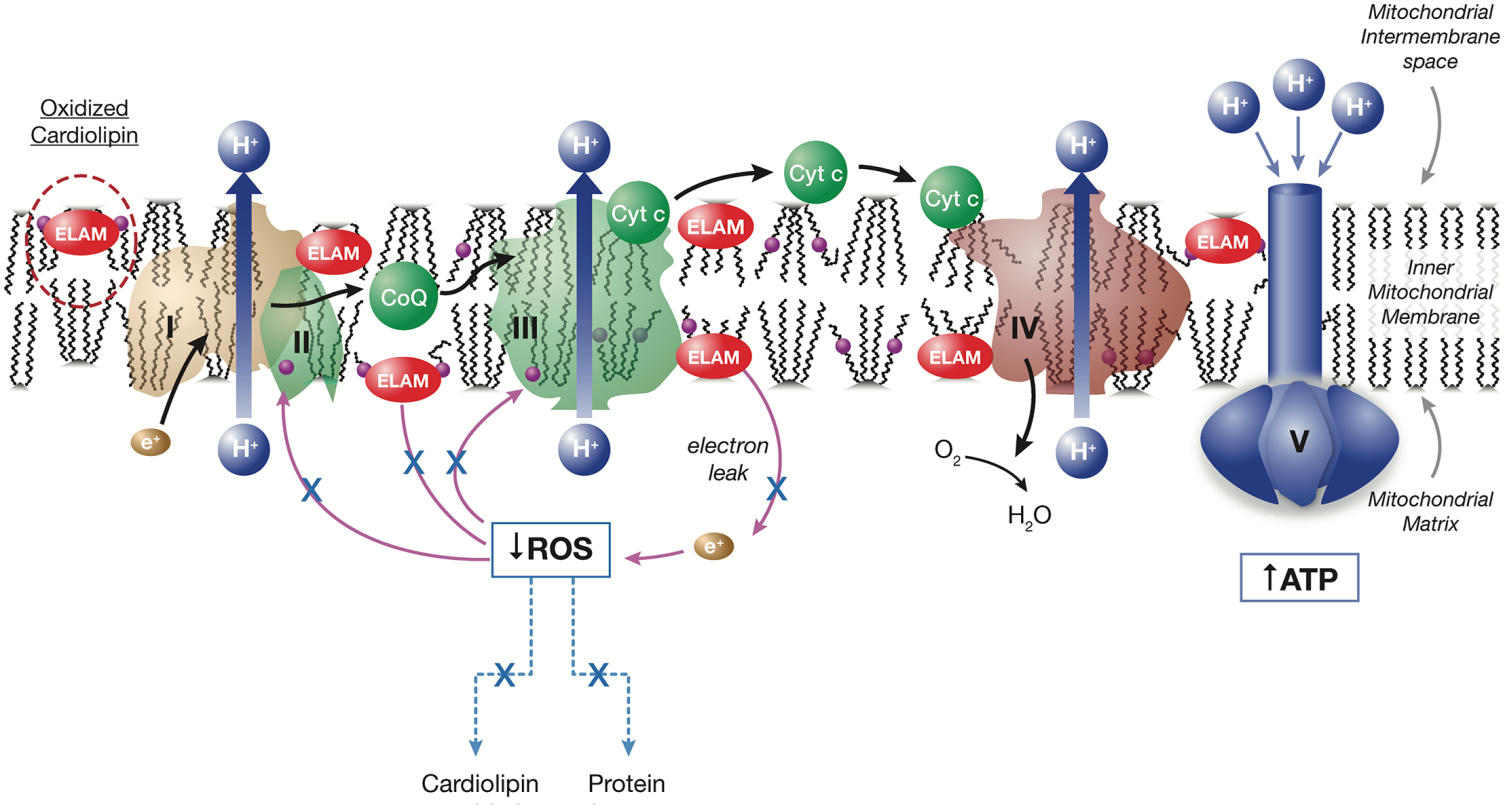
Elamipretide mechanism of action: Restoration of physiologically normal energetics. ATP = adenosine triphosphate, CoQ = coenzyme Q10, Cyt c = cytochrome C, ELAM = elamipretide, ROS = reactive oxygen species.

The cases presented depict the expanded‐access use of elamipretide in patients with ophthalmic symptoms secondary to diseases in which mitochondrial dysfunction plays a definitive role in disease etiology. The second case also provides insight regarding increasing the elamipretide dose in this patient population. CPEO plus and NARP syndrome are caused by genetic mutations that impact mitochondrial bioenergetics, and both disorders share several phenotypic traits related to mitochondrial dysfunction, as described in the case findings. Specifically, *MT‐ATP6* mutations block the generation of ATP, leading to the symptoms in NARP syndrome [8]. *POLG* mutations, which cause accumulation of errors in mtDNA during replication, result in impairment of the respiratory chain of the oxidative phosphorylation pathway and lead to the clinical symptoms of CPEO plus and other mitochondrial disorders [9]. Elamipretide has been proven to enhance ATP synthesis [5] and normalize oxidative phosphorylation in disease states [4, 10] as shown in Figure 1, supporting the use of this drug as a therapy for the disorders presented in this case series.

While elamipretide treatment has been evaluated in patients with PMM [6] and Barth syndrome [7], this drug has not been systematically studied in many other rare diseases associated with mitochondrial dysfunction. The EAP for elamipretide serves an important role in bringing a therapeutic candidate to these patients for whom no effective therapies are available. A small case series in patients receiving elamipretide through the EAP demonstrated that weight‐based dosing of elamipretide was well tolerated in pediatric patients with rare orphan mitochondrial diseases [11]. In this case series, a pediatric patient with 3‐methylglutaconic aciduria, deafness, encephalopathy, and Leigh‐like disease (MEGDEL syndrome) demonstrated similar improvements with elamipretide treatment, including developmental progress, lack of continued regression, and improvement in stamina and strength [11]. In another recently published case report, a patient with membrane protein‐associated neurodegeneration (MPAN) demonstrated symptomatic improvements in her dysarthria, dysphagia, gait, and functional outcome testing during elamipretide treatment [12]. Similarly, the two cases described in the present series support that elamipretide is generally well tolerated when given chronically as a 40–60 mg daily SC injection, with injection site reactions being the most frequently reported adverse event. Furthermore, the dramatic improvement in the conditions of both patients demonstrates the potential therapeutic benefit of elamipretide in diseases related to mitochondrial dysfunction, including potential ophthalmologic benefits that have yet to be clarified.

The initial 40 mg/day dose of SC elamipretide used in this case series has been extensively investigated in patients with a variety of mitochondrial diseases [6, 7]; however, little experience exists with patients on 60 mg/day doses. Efficacy and safety data presented from subjects with PMM [6] support 60 mg SC elamipretide as the current appropriate long‐term, daily dose to maximize benefits in patients with PMM. While improvement in symptoms was noted by both patients on doses of 40 mg/day SC elamipretide, additional benefits were observed with a dose increase to 60 mg/day in the patient with NARP syndrome. The 60 mg dose of elamipretide was well tolerated, and the injection site reaction occurrence was consistent with the 40 mg dose. This data may encourage dose titration in future patients enrolled in the EAP.

In conclusion, this case series provides evidence that the administration of 40–60 mg elamipretide SC injections was well tolerated and highlights the marked improvement associated with daily elamipretide therapy in patients with disorders related to mitochondrial dysfunction, including those with ophthalmic symptoms. This experience may guide the use of elamipretide in future patients enrolled in the EAP and help to inform clinical trial efforts.

## Author Contributions

**Sharique Ansari:** investigation, methodology, writing – original draft, writing – review and editing. **Mary Kay Koenig:** investigation, methodology, writing – original draft, writing – review and editing.

## Ethics Statement

FDA and local IRB review and approval were obtained prior to initiating therapy with elamipretide subcutaneously daily under this EAP.

## Consent

Written informed consent was obtained from the patients to be included in this study.

## Conflicts of Interest

The authors declare no conflicts of interest.

## Data Availability

The data that support the findings of this case report are not publicly available due to the data containing information that could compromise the privacy of the patient, but may be available from the corresponding author upon reasonable request.
